# Utilizing Digital Health to Collect Electronic Patient-Reported Outcomes in Prostate Cancer: Single-Arm Pilot Trial

**DOI:** 10.2196/12689

**Published:** 2020-03-25

**Authors:** Christine Tran, Adam Dicker, Benjamin Leiby, Eric Gressen, Noelle Williams, Heather Jim

**Affiliations:** 1 Thomas Jefferson University Sidney Kimmel Medical College and Cancer Center Jefferson Center for Digital Health & Data Science Philadelphia, PA United States; 2 Levine Cancer Institute at Atrium Health Southeast Radiation Oncology Group Charlotte, NC United States; 3 H Lee Moffitt Cancer Center Department of Health Outcomes and Behavior Tampa, FL United States

**Keywords:** eHealth, mHealth, smartphone, mobile phone, mobile apps, health promotion, chronic disease, health-related quality of life, cancer, patient-reported outcome measures, health information technology, patient-centered care

## Abstract

**Background:**

Measuring patient-reported outcomes (PROs) requires an individual’s perspective on their symptoms, functional status, and quality of life. Digital health enables remote electronic PRO (ePRO) assessments as a clinical decision support tool to facilitate meaningful provider interactions and personalized treatment.

**Objective:**

This study explored the feasibility and acceptability of collecting ePROs using validated health-related quality of life (HRQoL) questionnaires for prostate cancer.

**Methods:**

Using Apple ResearchKit software, the *Strength Through Insight* app was created with content from validated HRQoL tools 26-item Expanded Prostate Cancer Index Composite (EPIC) or EPIC for Clinical Practice and 8-item Functional Assessment of Cancer Therapy Advanced Prostate Symptom Index. In a single-arm pilot study with patients receiving prostate cancer treatment at Thomas Jefferson University Hospital and affiliates, participants were recruited, and instructed to download *Strength Through Insight* and complete ePROs once a week over 12 weeks. A mixed methods approach, including qualitative pre- and poststudy interviews, was used to evaluate the feasibility and acceptability of *Strength Through Insight* for the collection and care management of cancer treatment.

**Results:**

Thirty patients consented to the study; 1 patient failed to complete any of the questionnaires and was left out of the analysis of the intervention. Moreover, 86% (25/29) reached satisfactory questionnaire completion (defined as completion of 60% of weekly questions over 12 weeks). The lower bound of the exact one-sided 95% CI was 71%, exceeding the 70% feasibility threshold. Most participants self-identified with having a high digital literacy level (defined as the ability to use, understand, evaluate, and analyze information from multiple formats from a variety of digital sources), and only a few participants identified with having a low digital literacy level (defined as only having the ability to gather information on the Web). Interviews were thematically analyzed to reveal the following: (1) value of emotional support and wellness in cancer treatment, (2) rise of social patient advocacy in online patient communities and networks, (3) patient concerns over privacy, and (4) desire for personalized engagement tools.

**Conclusions:**

*Strength Through Insight* was demonstrated as a feasible and acceptable method of data collection for ePROs. A high compliance rate confirmed the app as a reliable tool for patients with localized and advanced prostate cancer. Nearly all participants reported that using the smartphone app is easier than or equivalent to the traditional paper-and-pen approach, providing evidence of acceptability and support for the use of remote PRO monitoring. This study expands on current research involving the value of digital health, as a social and behavioral science, augmented with technology, can begin to contribute to population health management, as it shapes psychographic segmentation by demographic, socioeconomic, health condition, or behavioral factors to group patients by their distinct personalities and motivations, which influence their choices.

**Trial Registration:**

ClinicalTrials.gov NC03197948; http://clinicaltrials.gov/ct2/show/NC03197948

## Introduction

### Prioritizing Patient-Reported Outcomes

As hospitals seek to provide better value in health care, patient-reported outcome measures (PROMs), such as those evaluating pain and distress, are an integral part of improving care. Patient-reported outcomes (PROs) can serve as an innovative way for providers to incentivize patients to make changes that facilitate patient engagement and self-care for chronic disease management and prevention [1-4]. The use of PROs for cancer patients is not only appropriate but also is becoming an increasing unmet need, as studies suggest remote monitoring has been evaluated and demonstrated to improve survival. Studies have shown that PROs can lead to improved patient communication with providers, engagement, satisfaction, and better health outcomes. PROs can enhance care management by understanding the impact of treatments on patients’ lives [5]. This is increasingly important in cancer care, as patients with cancer can experience changes in their nutrition, elimination, pain management, and sexual function at varying levels of severity [6]. A health-related quality of life (HRQoL) evaluation can be imperative to evaluate the results of clinical trials. However, there is substantial evidence that clinical investigators miss 40% to 74.4% of patients’ symptomatic adverse events (AEs) [7]. Moreover, major policy making entities have also emphasized the importance of incorporating PROs into cancer research and policy formation (including the National Cancer Institute [NCI], American Cancer Society, and US Food and Drug Administration). This interest reflects a growing national recognition that traditional medical outcomes (ie, survival and disease progression) do not fully capture the patient’s experience of health, and there is an unmet need for health care providers to capture a new definition of *value* of health care, which includes improvement in subjective outcomes of importance to patients [8]. The 2 principal methods of gathering PROs (before the advent of electronic methodologies) are paper-and-pen approach and clinician interview, both of which are labor and time intensive. For PROs to be routinely integrated into clinical practice, PRO data collection methods must be efficient by demonstrating convenient, instantaneous, inexpensive, reliable, and clinically feasible.

### A Digital Evolution

With the widespread adoption of smartphones, tablets, and other smart devices, mobile apps provide a new platform for patients to become active members of their health care team. Digital health technology encompasses clinical tools, advanced statistical algorithms, cloud computing, and artificial intelligence [9]. Research on digital technology can evaluate innovative approaches to improve care through PRO measures. The bidirectional transfer of data through smartphones offers an unprecedented method to collect PROs across the entire course of a cancer patient’s journey, allowing providers to monitor long-term outcomes. However, the translation of evidence-based health care interventions onto a digital platform should be evaluated to determine whether it is feasible and effective digital health technology [10-12].

The objective of this study was to test the feasibility and acceptability of *Strength Through Insight*, a digital health app collecting electronic PROs (ePROs) in patients with prostate cancer, and to examine patient perspectives to help create future digital health interventions. Apple’s ResearchKit empowers medical research by creating a mobile infrastructure for informed consent, surveys, and real-time active tasks (spatial memory, voice tests, motor activities, sleep-wake cycle, nutrition, and daily step counts) using the iPhone sensors and capabilities [13]. *Strength Through Insight*, a smartphone app built on the ResearchKit platform, aimed to explore the feasibility and acceptability of smartphone devices as a digital health tool to collect PROs for patients with cancer in the health care setting through a mixed methods approach.

To our knowledge, this is the first study piloting an ePRO using the ResearchKit smartphone app platform for patients with prostate cancer. The study (1) tested the feasibility of collecting ePROs via a digital health app, with a validated HRQoL questionnaires for patients undergoing prostate cancer treatment, and (2) analyzed patient attitudes and perceptions through qualitative interviews to identify reoccurring themes that address facilitators and barriers of adopting digital health to best support future design and implementation of digital health technology. We hypothesized that more than 80% of patients will complete 60% of the HRQoL questionnaires, once a week for a period of 12 weeks, proving the feasibility of ePROs in a smartphone app.

## Methods

### Procedures

This feasibility study was conducted at the Sidney Kimmel Cancer Center at Thomas Jefferson University Hospital (TJUH). Following TJUH institutional review board approval, potential participants were identified through a database maintained by the Sidney Kimmel Cancer Center.

The study was made available on the ClinicalTrials.gov [14] and on the Sidney Kimmel Cancer Center’s website in a listing of ongoing clinical trials. Potential participants were also recruited by research staff at TJUH in accordance with board-approved methods. Participating clinicians reviewed their patient lists and identified eligible patients who were then telephoned by a research staff member to confirm eligibility. Eligibility was confirmed by verbal confirmation of inclusion and exclusion criteria that were also available as part of the consent in the smartphone app. Eligible patients received detailed information regarding the study and, if interested, were sent a *next steps* document explaining possible risk and benefits, study expectations, expected time to complete, how to download the app, answer the questionnaire, configure settings (ie, cellular data and push notifications), set reminders, and encouraging reporting of their patient outcome via the HRQoL questionnaire. The document also included information on how to follow up with the research team if they felt that they needed additional support. Participants were required to provide informed consent for the study through their smartphones, on downloading the app, before answering any questions. All relevant information, including objectives of the study, required activities of participants, study procedures, confidentiality, and privacy of information, was provided in the consent. Patients with upcoming scheduled appointments at the cancer center had the option to meet with research staff during their clinic visit to get help downloading the app, configure app settings, or complete assessments.

The study investigators developed patient interview guide questions based on clinical experience and relevant literature. The guide consisted of questions regarding patients’ expectations and experiences throughout prostate cancer treatment, which was defined for participants as encompassing symptom management (perceived knowledge about treatment side effects), patient-physician communication, digital literacy, quality of life, social media, and patient satisfaction.

### Participants

Eligible patients were patients with prostate cancer who were receiving follow-up care (including follow-up and newly diagnosed patients). Inclusion criteria included (1) aged 18 years or older, (2) self-reported ability to speak and read English, (3) ability to communicate on a touch screen iPhone, (4) willing to provide signed informed consent, (5) willing and able to comply with all study activities, and (6) access to Wi-Fi connection or cellular data. Exclusion criteria included (1) subjects with concurrent medical or psychiatric condition who may have precluded participation in this study or completion of self-administered questionnaires (eg, moderate to severe dementia and/or severe, uncontrolled schizophrenia or other conditions that would render them unable to complete a questionnaire) and (2) cognitive or other impairment (eg, visual) that would interfere with completing a self-administered questionnaire on an iPhone.

### Measures

The NCI Symptom Management and Health-Related Quality of Life Steering Committee recommend core sets of PROs to be routinely incorporated in prostate cancer treatment. This effort suggested 5 domains for localized prostate cancer (urinary incontinence, urinary obstruction and irritation, bowel-related symptoms, sexual dysfunction, and hormonal symptoms) and 4 domains for advanced prostate cancer (pain, fatigue, mental well-being, and physical well-being) [6]. Participants were stratified based on the severity of clinical diagnosis and asked to answer questionnaires that incorporated the recommended domains and comprehensively covered the multiple areas related to HRQoL. Patients with localized prostate cancer were asked to answer weekly questions from *survey A*, which included the validated HRQoL tool, 26-item Expanded Prostate Cancer Index Composite (EPIC-26; urinary incontinence and irritation/obstruction items, along with bowel, sexual, and vitality/hormonal domains), and patients with advanced prostate cancer were asked to answer weekly questions from *survey B*, which included the validated HRQoL tools Expanded Prostate Cancer Index Composite for Clinical Practice (EPIC-CP) and 8-item Functional Assessment of Cancer Therapy Advanced Prostate Symptom Index (FAPSI-8; urinary incontinence and irritation/obstruction items, along with bowel, sexual, and vitality/hormonal; pain; fatigue/lack of energy; weight loss; and worry domains). The research team determined the frequency of completing the assessment as once per week over a period of 12 weeks.

**Figure 1:**
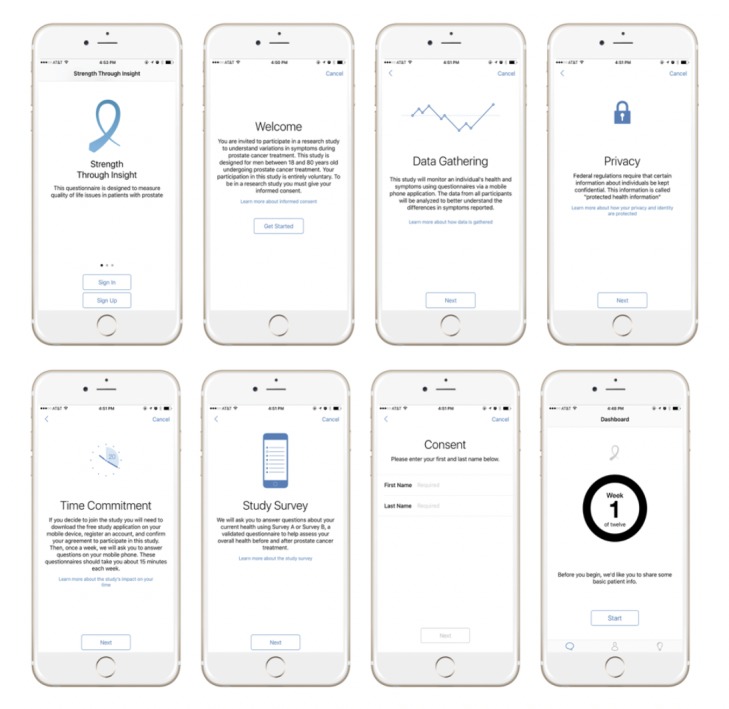
Screenshot representations of the *Strength Through Insight* app.

Participants were asked to complete the EPIC demographic add-on survey in the smartphone app, which included information on the participant’s ethnicity, marital status, employment, smoking status, previous treatments for prostate cancer, other current medical conditions, education, and income. Participants were then asked to self-select a survey option (survey A or survey B) based on the stage of their cancer [6]. Patients with localized prostate cancer were asked to answer weekly questions from *survey A*, which included the validated HRQoL tool, EPIC-26 (urinary incontinence and irritation/obstruction items, along with bowel, sexual, and vitality/hormonal domains), and patients with advanced/metastatic prostate cancer were asked to answer weekly questions from *survey B*, which included the validated HRQoL tools EPIC-CP and FAPSI-8 (urinary incontinence and irritation/obstruction items, along with bowel, sexual, and vitality/hormonal; pain; fatigue/lack of energy; weight loss; and worry domains). Patients were then asked to commit to an estimated 10 to 15-min participation in the study and configured app settings to allow or deny push notifications to serve as study reminders. The research team determined the frequency of completing the assessment as once per week over a period of 12 weeks. Throughout the study’s duration, all participant information was deidentified and stored using the secure, Health Insurance Portability and Accountability Act compliant, CloudMine data repository. Screenshots of the *Strength Through Insight* app are shown in Figure 1.

The feasibility of the *Strength Through Insight* app was determined by a dichotomous measure of satisfactory completion (defined as 60% of weekly questions over a 3-month period). The rate of satisfactory completion was estimated along with a 1-sided exact 95% CI. The method is considered feasible if the lower bound of the CI is above 0.7. Acceptability of *Strength Through Insight* was determined through patient opinions, regarding ease of use, satisfaction, and impact on cancer care.

### Patient Interviews

Participants were asked to take part in guided interviews via telephone or in-clinic during visits before completing their first assessment. Separate interviews were held at the end of the study to facilitate feedback on *Strength Through Insight* and open discussion of topics, such as symptom management, attitude toward ePRO collection, or preferred method of reporting to elicit patient opinion on acceptability. Participants were asked to self-report their digital literacy as having a high digital literacy level (defined as the ability to use, understand, evaluate, and analyze information from multiple formats from a variety of digital sources) or low digital literacy level (defined as only having the ability to gather information on the internet). Interviews were conducted by a moderator who had previously received research training from an experienced qualitative health teacher. Moderators were not affiliated with the patient’s oncology care team. Content analysis of interviews provided common themes, illustrating the informational needs and concerns regarding remote PROs and digital health technology.

Pre- and postinterview questionnaires are provided in Multimedia Appendices 1 and 2.

## Results

### Participants

A total of 29 patients with prostate cancer participated in the study from August 1, 2016, to December 31, 2017. The main reason for ineligibility was because of owning an Android device, and common causes for participant refusal were unwillingness to download the app, unable to remember iOS password (required to download apps), lack of knowledge concerning apps, and concern of data security. As shown in Table 1, the median age of participants was 55 years (range 45-70 years). The majority of participants were white, married, completed college or beyond, digital literacy of Health 2.0, and reported a current annual household income of US $30,001 to US $100,000. Overall, 26 (89%) reported a diagnosis of localized prostate cancer.

**Table 1:** Sociodemographic and clinical characteristics of participants (N=29).

Characteristics		Values
Age (years), median (range)		55 (45-70)
**Race, n (%)**		
	White	29 (100)
**Marital status** **, n (%)**		
	Married	19 (66)
**Education, n (%)**		
	College	29 (100)
**Annual household income, n (%)**		
	≥$30,000	29 (100)
**Diagnosis, n (%)**		
	Localized prostate cancer	25 (86)
	Advanced prostate cancer	4 (14)
**Digital health literacy level, n (%)**		
	High	8 (27)

### Measures

#### Quantitative Evaluation: Feasibility (Assessment of Satisfactory Completion)

A total of 29 participants enrolled in the study. Of these 86% (25/29) reached satisfactory questionnaire completion (defined as completion of 60% of weekly questions over 12 weeks). The lower bound of the exact 1-sided 95% CI was 71%, exceeding the 70% feasibility threshold. All participants were able to complete informed consent through the smartphone app. Patterns of missing data showed a decline in responses after week 6. Moreover, 3 participants reported technical issues (app randomly closing/needing to restart the app) as the main reason for missing questions. Of 29, 90% (26/29) participants self-identified with localized disease chose to opt in push notifications. These participants reported push notifications to be an effective tool as a passive reminder to monitor their health. However, patients with advanced disease preferred not to have push notifications because of the patient belief that the notifications served as reminders of their health status or poor quality of life.

#### Qualitative Evaluation: Acceptability (Use, Satisfaction, and Impact on Care)

Overall, 72% (21/29) involved in testing the feasibility of the *Strength Through Insight* app were a part of the guided interviews. Representative patient quotes are presented in Textbox 1. All patients reported that using *Strength Through Insight* is easy or equivalent compared with completing a paper copy of the questionnaire. Most participants cited text messaging as their preferred method of reporting symptoms. Although most patients did not express a preferred recall or frequency to report symptoms (cited wanting to report symptoms when they experience side effects and not by an unpredictable time point), several participants expressed bother in reporting symptoms too frequently. Reasons for skipping weekly questionnaire included lack of adequate time in personal schedules, lack of perceived value, technical problems, and issues with frequency/recall. Although no patients recalled discussing their assessment report during clinic visits, patients reported that an increase in personal awareness of symptoms facilitated increased communication with caregivers, families, and friends rather than providers. Participants expressed a desire for more personalized questions and noted skipping questions if symptoms did not apply, suggesting the need for identifying a patient’s high priority concerns and symptoms to reduce the burden on patients completing questionnaires.

**Representative participant responses to interview questions.**

- “Every time I saw the notification on my phone, even if I didn’t automatically go to the app and answer the questionnaire, it made me think of how I’m doing. And if I had a question about something I was feeling, I go to Dr. Google and search for my symptoms and look at forums from people to see if I can find someone like me.”
- “I thought it was great! I would be interested on using it for the rest of my treatment because you can’t remember everything, it’s hard to bring up anyways because my visits are so fast paced. I think my doctor gets really defensive every time I come in with my sheets.”
- “My wife and I actually answered the questions together as a little ritual at the end of the week. We had long talks about some of it because she made me change my answers to some questions.”
- “Some of the questions were just too general or repetitive. It wasn’t specific to me and I wish there were more questions about my pain management. I had issues that were worsened by surgery.”

### Patient Interviews

Analysis of interview data revealed 4 dominant digital health themes: (1) the value of emotional support and wellness in cancer treatment, (2) rise of social patient advocacy in online patient communities (OPCs) and networks, (3) concerns over privacy and privacy as a social norm, and (4) the need for personalized digital health to improve patient engagement.

#### Theme 1: The Value of Emotional Support and Wellness in Cancer Treatment

Analysis revealed repeated reference to how *Strength Through Insight* and similar apps are fueled by questions specifically relating to symptoms and AEs and either do not include or only briefly discuss wellness. Most participants mentioned a lack of confidence and awareness in responding to questions outside disease and treatment and criticized apps for undermining the value of emotional support. A few participants specifically noted their desire to invest in mental and emotional well-being more than tending to treatment symptoms was emphasized. Similar interview responses pointed to the lack of questions focused on psychosocial or emotional support in the self-management of cancer treatment.

#### Theme 2: The Rise of Social Patient Advocacy in Online Patient Communities and Networks

The particular role of social media through OPCs and networks was emphasized as a recurring topic. Several participants identified the use of patient communities, such as Facebook groups, the smartphone app *Belong*, or content communities, such as Reddit, as the main environment to obtain trusted information and connect with other cancer patients and caregivers. Some patients reported the use of online communities over education websites, such as WebMD. In addition, a few patients reported the preference of these platforms over clinic visits with providers because fellow cancer patients are seen as experts and offer *more and better information*.

#### Theme 3: Concerns Over Privacy as a Social Norm

Nearly all participants emphasized a concerning issue of how patient-generated data would be used in the future. Participants identified the lack of transparency regarding current and future use of data as a major concern. Several participants also noted that despite potential disagreement on how information will be used or clarity of data ownership, they are likely to consent to health apps regardless because they will not be able to benefit from digital health technology otherwise.

#### Theme 4: The Key to Driving Patient Engagement: Personalization

Almost all patients reported predictive information as an encouragement to participate in their health care and acknowledged a desire for the app to be supported through predictive analytics to help drive engagement and healthy behavior change. Several participants specifically asked for an app upgrade that included a data summary component, showing data analytics to capture a higher level of detail necessary to predict and personalize symptoms if asked to continue app use. A summary of the qualitative themes has been illustrated in Textbox 2.

**Summary of qualitative themes.**

**Theme 1: The value of emotional support/wellness in cancer treatment**

- “I’d rather an app pay attention to how I’m doing emotionally. Weeks go by and I still end up sleeping the day or weekend away because I’m depressed and tired. I avoided seeing anyone and used symptoms I didn’t even have as an excuse.”
- “I don’t know if I was depressed. I’m a proud person and didn’t let anyone know how I was feeling really. But my doctor didn’t even ask. Maybe they need input from other departments.”

**Theme 2: The power online patient communities and networks**

- “I’ve changed doctors three times, so I know I like my doctor. But I was disappointed because he made me feel confident that I would only experience certain symptoms. Then 3-4 months down the road, I never thought I would have the pain or some side effects I have now. I wasn’t told of anything really.”
- “I go on there because I’m interested and want to know more and those are the people I want to talk to. They make me feel like I can take my life back and move on.”

**Theme 3: Privacy as a social norm**

- “It’s hard to trust. We don’t have the same type of security on the internet than we do in the real-world. Especially with companies, I don’t believe they have my best interest in mind. I don’t see my opinion changing.”

**Theme 4: Desire for personalized patient engagement**

- “Without some kind of data analysis, I feel like I’m just the product giving you information, not the consumer.”

## Discussion

### Digital Health: Personalizing Health Care

The aim of this study was to explore the feasibility and acceptability of collecting ePROs using validated HRQoL assessment tools through a smartphone app in adult men throughout their course of treatment for prostate cancer. This study found that 86% (25/29) of participants reached satisfactory questionnaire completion (defined as completion of 60% of weekly questions over 12 weeks), proving the feasibility of collecting ePROs through a digital health app. Patients reported skipping domain-specific questions (urinary incontinence and irritation/obstruction items, along with bowel, sexual, and vitality/hormonal domains) that did not apply to the particular individual. Although higher completion rates over time would be desirable, the study was unable to observe this. This may be because of the lack of feedback to the user regarding their previous answers to the questionnaire. In this regard, the study did not originally consider patient feedback and was one of the learning points the authors were able to extract from this study. Overall, the use of ePROs may improve the quality of routine cancer care by expediting the detection of severe or disabling toxicities [2,15]. Although patients reported facilitated communication between patients and caregivers, the lack of increased communication between patient and provider suggests the requirement for an educational support tool. Patients may need to be educated on the best practices of self-monitoring and management of cancer to understand how to manage lifestyle choices to improve outcomes. This is consistent with other studies on patient empowerment in prostate cancer, which identified the need for provider support for the self-management of prostate cancer and social networks as an important resource that could be integrated into interventions [16,17].

### Patient Acceptance

A primary theme that emerged from the interviews emphasized the value of emotional support and wellness in cancer treatment. Patient interviews highlighted the importance of emotional well-being as an unaddressed side effect of cancer treatment that is dealt with every day, as opposed to the appearance of occasional symptoms. This demonstrates another impactful way digital health can deliver care to meet a patients’ need within an empowerment framework.

A second major theme was concerned with the importance of providers to engage patients in participatory medicine with shared decision making. As a result of this lack of engagement, patients have resorted to social networking platforms such as OPCs. OPCs and networks are known for the arrangement and abundance of information, which enable patients to make treatment decisions that correspond with their long-term goals [18]. This research adds evidence to the emerging trend of community-based social media platforms as a common way for patients to self-manage their health conditions [19]. Social cognitive theory along with social network analysis suggests patients are influenced by OPCs because of the social support received from online peers and a patient’s self-reflection. For example, in a recent qualitative pilot study, a Facebook support group was created for liver transplant patients to use in a virtual community forum. The study examined the effect of the OPC on patient engagement and demonstrated an overall positive impact on patient care, and the main motivation for joining the group was reported to provide or receive support from other patients [19]. In a poststudy survey, patients cited their primary reason for participating in the Facebook group was to provide and receive support from other patients [19].

A third major theme that emerged puts forward an important fundamental question in digital health and how patients can benefit from data while protecting their privacy. Patient concerns over privacy, confidentiality, and control of data represent a growing recognition that patient-generated data from digital health tools can potentially be used for wanted and unwanted outcomes. Without public policy regulation concerning the ownership and responsibility for patient-generated data, digital health continues to lack transparency over the control of data, along with its implication for advanced analytics [20,21]. For example, several smartphone apps’ default option benefits data collection by effectively setting the default to *opt-in* rather than *opt-out*, granting apps’ access to sensors and data to collect private information (location data, Web browser history, and photos).

A fourth major theme indicated the inefficiency of digital health interventions unless appropriately acted on by patients. Nearly all patients reported a desire to become more involved with their health care through data personalization and predictive analytics. Lessons learned from the compliance rate of *Strength Through Insight* suggest that by combining objective measures of disease while incorporating the perspective of the patient, predictive analytics could increase participation. This requires digital health apps to convert patient-generated data into a functioning algorithm that factors and combines data elements to produce a useful prediction [22,23]. ePROs that include personalized reporting measures with symptomatic and psychometric properties integrated into prediction models can potentially deliver faster more accurate insights to support medical decision making [24,25]. The lack of patient engagement is a specific challenge that prevents ePROs from being integrated and used in decision making. As researchers use digital health to leverage advanced machine learning algorithms, digital health interventions should also be used to educate patients to better understand and control their own risk and learn how to appropriately act on implications provided in personalized statistics [26]. Patients, as consumers of health care, are the ultimate users and stakeholders of digital health technology, and future research must identify ways to best engage patients and caregivers.

A limitation of the study included the lack of a diverse patient population, which may not be representative of all patients with prostate cancer. The limited population may be reflected of a *digital divide* because most participants were self-reported to be White, educated, wealthier, and with a high digital literacy. The requirement of an iOS device also made several interested patients ineligible because of technology restrictions. The app was also built before Apple’s most recent announcement for the new capability of iOS 11.3 in CareKit, which enables patients to download laboratory results, allergies, immunizations, medications, procedures, and vitals from hospitals. Other limitations to this study may a higher level of patient motivation from the participants compared with the average patient, in which these patients were more personally inspired to complete the tasks. This may have also led to a secondary bias in the patient’s choice to participate in the study interviews, as previous studies have suggested that prostate cancer patients may be more compliant with PROs than other cancer patients who have more complex care (eg, head and neck cancer patients). Finally, the study used the validated HRQoL tools EPIC-26, EPIC-CP, and FAPSI-8, which were validated to be used on a monthly basis, and Strength Through Insight app’s mode of asking patients to answer questionnaires on a weekly basis instead.

### A Path Forward

In the last few years, the health care industry has been promoting the concept of providers and patients collaborating and communicating with each other as a powerful tool. This has led to an evolving model of consumerism and a desire of patients to become engaged in their own health care decisions, delivery, and interactions. As the digital era progresses, digital health may serve as an enabler of patient-provider engagement, extending care beyond the confines of the hospital system and meeting consumers on their own terms. Digital platforms can engage consumers in a variety of ways, including tracking medical progress, treatment adherence, reminders and scheduling, and communications and providing the ability to capture more comprehensive data for analysis. However, despite the benefits of desire, many digital health technologies face the challenge of personalization, as health care has historically taken a one size fits all approach to patient engagement using the same context and communication channel for every patient. Future technology must focus on each patient as a unique individual, with his or her own motivations, priorities, and communication preferences. Moreover, the health care industry has the opportunity to improve on traditional mass approaches to patient communication by leveraging lessons learned in both the retail and financial industries [27,28]. Future digital health apps should not only focus on the development of technology but use health behavior and belief models to facilitate design techniques that incorporate patient perspectives to prompt behavior change.

### Conclusions

The health care community has long recognized the value of a patients’ input in describing their own experiences, which has led to the growing use of ePROs to improve the efficiency of data collection and provide new opportunities to bring meaningful evidence back to patients and providers in innovative ways. This research provided an in-depth perspective on the different aspects of implementing ePROs on a digital health platform. To our knowledge, this was the first study piloting an ePRO using the ResearchKit smartphone app platform for patients with prostate cancer and expands on the research proving the feasibility and rigor of ePROs. With this, the true value of digital health, as a social and behavioral science, augmented with technology, can begin to contribute to population health management, as it shapes psychographic segmentation by demographic, socioeconomic, health condition, or behavioral factors to group patients by their distinct personalities and motivations, which influence their choices.

## Appendix

Multimedia Appendix 1 Preinterview questions.

Multimedia Appendix 2 Postinterview questions.

## Abbreviations

AE: adverse event
EPIC-26: 26-item Expanded Prostate Cancer Index Composite
EPIC-CP: Expanded Prostate Cancer Index Composite for Clinical Practice
ePRO: electronic patient-reported outcome
FAPSI-8: 8-item Functional Assessment of Cancer Therapy Advanced Prostate Symptom Index
HRQoL: health-related quality of life
NCI: National Cancer Institute
PRO: patient-reported outcome
TJUH: Thomas Jefferson University Hospital
