# Supplementary figures and images for: Utilizing Digital Health to Collect Electronic Patient-Reported Outcomes in Prostate Cancer: Single-Arm Pilot Trial
Source: J Med Internet Res. 2020 Mar 25;22(3):e12689. doi: 10.2196/12689 (PMC7142743; doi:10.2196/12689)

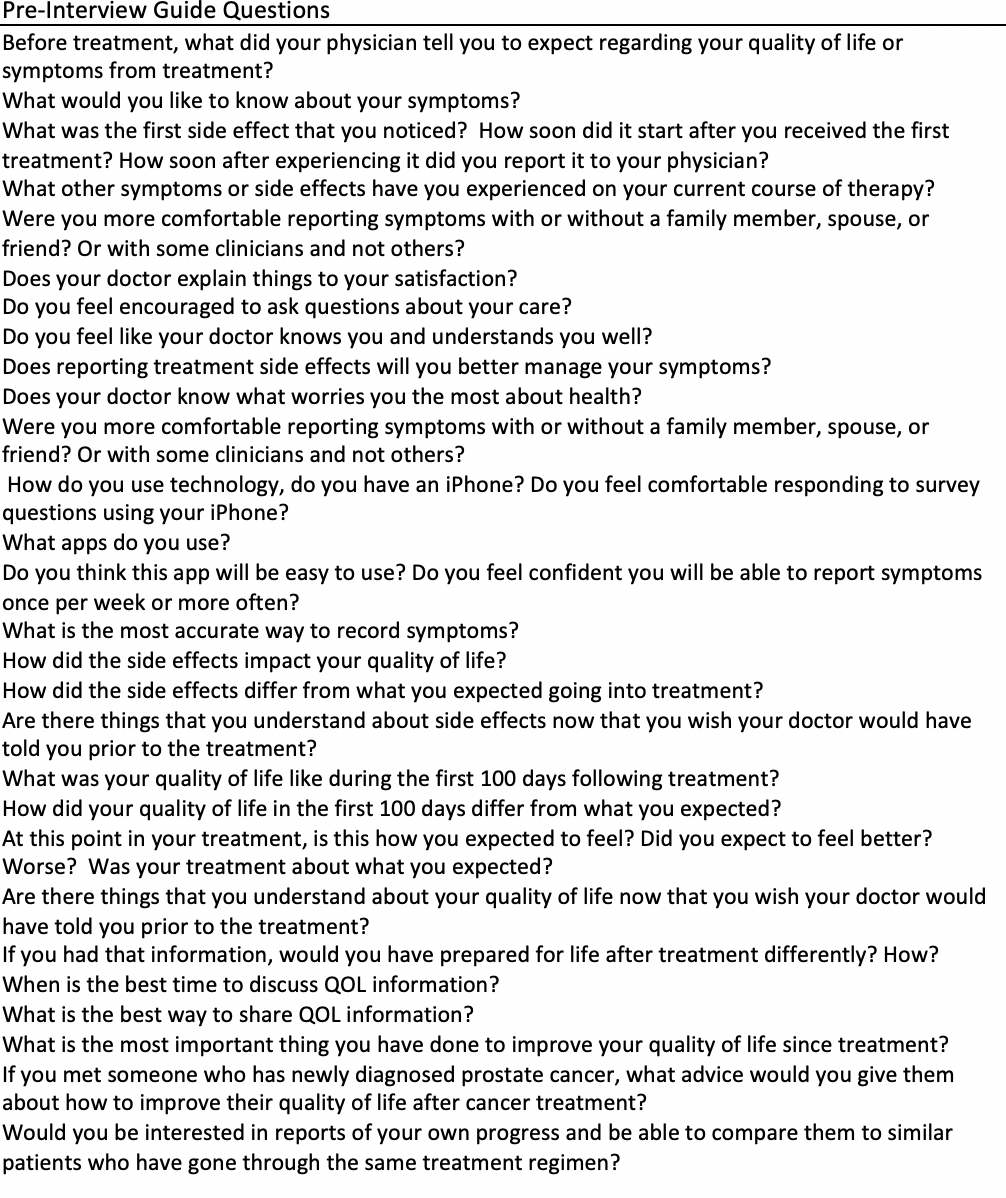

Supplement: Multimedia Appendix 1 [file jmir_v22i3e12689_app1.png]

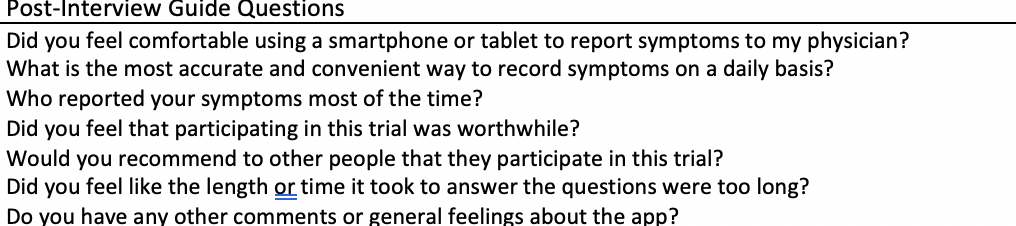

Supplement: Multimedia Appendix 2 [file jmir_v22i3e12689_app2.png]
